# Head and neck cancer in Australia between 1982 and 2005 show increasing incidence of potentially HPV-associated oropharyngeal cancers

**DOI:** 10.1038/sj.bjc.6606091

**Published:** 2011-02-01

**Authors:** J S Hocking, A Stein, E L Conway, D Regan, A Grulich, M Law, J M L Brotherton

**Affiliations:** 1 Centre for Women's Health, Gender and Society, University of Melbourne, 2/723 Swanston Street, Carlton 3053, Victoria, Australia; 2 CSL Limited, 45 Poplar Road, Parkville 3052, Victoria, Australia; 3 National Centre in HIV Epidemiology and Clinical Research, University of New South Wales, Building CC4, 45 Beach Street, Coogee 2034, New South Wales, Australia; 4 National Centre in HIV Epidemiology and Clinical Research, Ground floor, CFI Building, Corner Boundary and West Streets, Darlinghurst 2010, New South Wales, Australia; 5 National Centre in HIV Epidemiology and Clinical Research, University of New South Wales, Building CC4, 45 Beach Street, Coogee 2034, New South Wales, Australia; 6 National HPV Vaccination Program Register, 1/250 Victoria Parade, East Melbourne 3002, Victoria, Australia

**Keywords:** oropharyngeal cancer, human papillomavirus, oral cavity cancer

## Abstract

**Background:**

Although tobacco- and alcohol-associated head and neck cancers are declining in the developed world, potentially human papillomavirus (HPV)-associated oropharnygeal cancers are increasing.

**Methods:**

We analysed oropharyngeal and oral cavity cancer rates in Australia in 1982–2005. Cancers from the oropharynx (base of tongue, tonsil and other specific oropharyngeal sites) were classified as potentially HPV associated (*n*=8844); cancers in other oral cavity and oropharyngeal sites not previously associated with HPV were classified as comparison (*n*=28 379).

**Results:**

In 2000–2005, an average of 219, 159 and 110 cancers of the tonsil, base of tongue and other oropharyngeal sites were diagnosed annually, with incidences of 1.09 (95% CI: 1.03, 1.15), 0.79 (95% CI: 0.74, 0.84) and 0.55 (95% CI: 0.50, 0.59) per 100 000, respectively. An average of 1242 comparison cancers were diagnosed annually (6.17 (95% CI: 6.03, 6.31) per 100 000). In 1982–2005, there were significant annual increases in tonsil (1.39% (95% CI: 0.88, 1.92%)) and base of tongue cancers in males (3.02% (95% CI: 2.27, 3.78%)) and base of tongue cancer in females (3.45% (95% CI: 2.21, 4.70%)). There was a significant decrease in comparison cancers in men (−1.69% (95% CI: −1.96, −1.42%)), but not in females.

**Conclusion:**

Potentially HPV-associated oropharyngeal cancer in Australia is increasing; the impact of HPV vaccination on these cancers should be monitored.

Cancers of the head and neck arising from the mucosa lining the oral cavity, oropharynx, hypopharynx, larynx, sinonasal tract and nasopharynx (Marur *et al*, 2010) represent a considerable burden worldwide, being the fifth most common cancer in 2008 (IARC, 2010). Tobacco use and alcohol consumption are known risk factors for many of these cancers (Blot *et al*, 1988; Franceschi *et al*, 1990; Hashibe *et al*, 2007), but more recently human papillomavirus (HPV) infection has been found to be strongly associated with oropharyngeal cancer (Gillison *et al*, 2000; Herrero *et al*, 2003; Gillison, 2004).

There are epidemiological differences between HPV-DNA-positive and HPV-DNA-negative head and neck cancers, HPV-DNA-positive cancers are associated with younger age and higher numbers of sexual partners, but are less associated with tobacco smoking compared with HPV-DNA-negative cancers (Chaturvedi *et al*, 2008; Gillison *et al*, 2008; Heck *et al*, 2010). The proportion of head and neck cancers that are HPV DNA positive varies considerably. The most recent systematic review found that the average HPV DNA positivity was 35.6% for oropharyngeal cancer and 23.5% for oral cavity cancer (Kreimer *et al*, 2005). However, this review classified all tongue cancers as oral cavity cancers and did not differentiate between base of tongue (classified anatomically as an oropharyngeal site) and surface or border of tongue (classified anatomically as an oral cavity site; Kreimer *et al*, 2005). As HPV DNA positivity varies by site and is highest in the tonsil and base of tongue (Herrero *et al*, 2003; Gillison, 2007), this review may have underestimated oropharyngeal cancer HPV DNA positivity and overestimated oral cavity cancer positivity. This review also showed that there is substantial variation in the proportion of HPV-DNA-positive cancers by country and study; part of this may be because of the different distributions of risk factors other than HPV infection such as tobacco consumption and also to the accuracy of cancer site classification.

Although the incidence of head and neck cancers associated with tobacco and alcohol consumption has decreased considerably in the developed world, that of oropharnygeal cancers has increased (Ryerson *et al*, 2008; Chaturvedi *et al*, 2009). Given the aetiological role of HPV in some oropharyngeal cancers, it is possible that the incidence of these cancers may decline after HPV vaccination. In this report, we examine the incidence of oropharyngeal and oral cavity cancer in Australia from 1982 to 2005.

## Methods

Cancer incidence data were obtained from the National Cancer Statistics Clearing House database (NCSCH), which was established in 1986 to co-ordinate cancer statistics nationally at the Australian Institute of Health and Welfare. It collates data provided from the eight Australian State and Territory Cancer Registries, covering the entire Australian population.

Cancer cases were coded according to the version of the International Classification of Disease for Oncology (ICD-O) that was in use at the time of diagnosis. The third edition of the ICD-O (ICD-O-3) was used for diagnosis years from 2001 onwards; the data using the previous editions were converted to ICD-O-3 codes using pre-defined and standard mapping algorithms. The Registries use the International Agency for Research on Cancer (IARC) and the International Association of Cancer Registries rules to code all cancer diagnoses (Fritz *et al*, 2000; IARC, 2004). For the purpose of this analysis, and consistent with other published similar studies (Ryerson *et al*, 2008), cancers arising in the following oropharyngeal sites were classified as ‘potentially HPV associated’ – base of tongue and lingual tonsil, tonsil and Waldeyer's ring and other specific sites within the oropharynx. Cancers in other oral cavity and oropharyngeal sites that have not been previously associated with HPV infection were also included in the analysis and classified as ‘comparison’ site cancers (Ryerson *et al*, 2008). (Table 1) All analyses were restricted to squamous cell histologies (ICD-O-3 codes 8500 to 8076, 8078, 8083, 8084 and 8094). The number of cancer cases diagnosed between 1 January 1982 and 31 December 2005 were obtained stratified by age group (five year age groups), sex, ICD-O code and histology.

### Statistical analyses

Age-specific incidence rates were calculated by year of cancer diagnosis and by birth cohort. Birth cohorts were generated by subtracting the midpoint of attained age from the year of cancer notification, and regrouped in 5-year birth cohort periods. Incidence rates were age standardised to the Australian 2001 standard population (Australian Bureau of Statistics, 2008), and their trends by year of cancer diagnosis were analysed using generalised linear models with a logarithmic link function and expressed as annual percentage change in incidence. Goodness of fit of the model was checked using the Pearson statistic and the reported models fit the data well (*P*>0.990 for all models). Trends in mean age over the study period were examined using linear regression models. Model fit was checked using standard diagnostic plots and the normal distribution of residuals was confirmed using the Shapiro–Wilks test.

All analysis was undertaken using Stata Version 11.0 (StataCorp, 2010).

## Results

**Figure 1:**
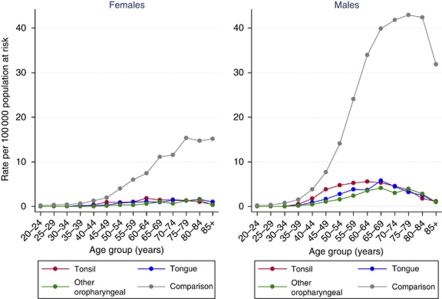
Age-specific incidence rates by gender and cancer site, 2000–2005.

During the study period from 1982 to 2005, 8844 cases of potentially HPV-associated oropharyngeal cancers were diagnosed, of which 44.1% were tonsillar, 29.5% were base of tongue and 26.4% were other oropharyngeal cancers, and 28 379 cases of comparison site cancers were diagnosed. The age-adjusted incidence of both potentially HPV-associated and comparison cancers was consistently three- to fourfold higher among males than females (Table 1). Potentially HPV-associated oropharyngeal cancers were diagnosed at younger ages on average than comparison site cancers (59.8 *vs* 64.4 years for males in 2005 (*P*<0.01) and 63.6 *vs* 66.9 years for females in 2005 (*P*<0.01)). The age at diagnosis for potentially HPV-associated oropharyngeal cancers decreased significantly each year by 0.06 years (95% CI: 0.01, 0.11) for males with no change for females. In males, the overall annual decrease in the age at diagnosis reflected a significant decrease of 0.18 years (95% CI: 0.11, 0.24) for tonsillar cancers and no significant change in age at diagnosis of cancers of base of tongue or other oropharyngeal sites. The age at diagnosis for comparison site cancers increased significantly each year in both males and females (Table 2). During the period 2000–2005, the age-specific incidence of potentially HPV-associated cancers was highest among those aged 60–69 years at diagnosis compared with 70–79 years for comparison site cancers (Figure 1).

**Figure 2:**
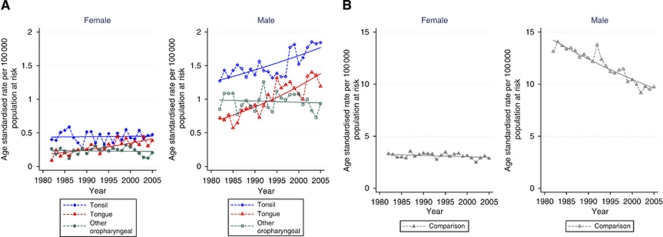
Age standardised incidence trends by calendar year of diagnosis for potentially HPV-associated oropharyngeal cancers (squamous cell carcinomas; (**A**) and comparison site cancers (squamous cell carcinomas; (**B**), 1982–2005.

The incidence of potentially HPV-associated cancers increased significantly for both males and females during the study period with an annual percentage increase of 1.04% (95% CI: 0.40, 1.68%) for females and 1.42% (95% CI: 1.08, 1.76%) for males. The increase was greatest for base of tongue for both males and females with increases of >3% per year. The incidence of comparison site cancers decreased significantly for males with rates remaining relatively stable for females (Table 2 and Figure 2). The proportion of cancers diagnosed each year at potentially HPV-associated oropharyngeal sites increased from 18.60% in 1982 to 28.70% in 2005 (*P*<0.01).

**Figure 3:**
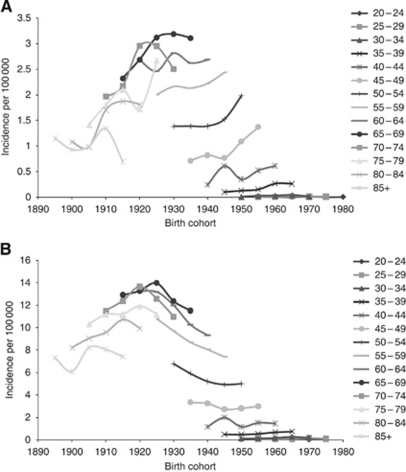
Incidence trends by cohort year of birth for potentially HPV-associated oropharyngeal cancers (squamous cell carcinomas; (**A**) and comparison site cancers (squamous cell carcinomas; (**B**), Australia.

Potentially HPV-associated oropharyngeal cancers showed a general pattern of higher incidence among recent compared with distant birth cohorts. Among the age groups 45–49 years, 50–54 years and 55–59 years, incidence rates were lowest for the most distant birth cohorts (cohorts born between 1925 and 1940); incidence increased thereafter for each successive cohort and peaked for cohorts born between 1945 and 1955. In contrast, age-specific incidence rates for comparison cancers showed a general pattern of decreasing incidence among recent compared with distant birth cohorts (Figure 3).

## Discussion

Within Australia, the incidence of potentially HPV-associated oropharyngeal cancers increased between 1982 and 2005, and was more marked within recent birth cohorts. The incidence of comparison site cancers declined significantly during the same period, particularly among recent birth cohorts. The proportion of cancers occurring that are potentially HPV-associated increased significantly during the study period as a result of both decreasing incidence of comparison site cancers and increasing incidence of potentially HPV-associated oropharyngeal cancers. Both potentially HPV-associated oropharyngeal and comparison site cancers were predominantly diagnosed in males. In addition, the age at diagnosis for potentially HPV-associated oropharyngeal cancer declined significantly over time for males, consistent with the reported younger age for individuals with HPV DNA-positive *vs* HPV DNA-negative oral cavity and oropharyngeal cancer (Gillison, 2007; Hong *et al*, 2010).

Our findings are consistent with previous studies of trends in oral cavity and oropharyngeal cancers (Shiboski *et al*, 2005; Sturgis and Cinciripini, 2007; Chaturvedi *et al*, 2008; Ryerson *et al*, 2008), and further supported by studies that have examined the proportion of oropharyngeal cancers that is HPV DNA positive. In Sweden, the proportion of cancer of the base of tongue that was HPV DNA positive increased from 58% during 1998–2001 to 84% in 2004–2007 (Attner *et al*, 2010) and the proportion of tonsillar cancer that was positive increased from 68% in 2000–2002 to 93% in 2006–2007 (Nasman *et al*, 2009). A case series conducted in Australia showed that the proportion of oropharyngeal cancer that was positive increased from 19% in 1987–1990 to 66% in 2005–2006 (Hong *et al*, 2010). In each case, over 80% of the HPV-DNA-positive cancers contained HPV16.

It is likely that our findings are real effects, because the procedures for diagnosing, reporting and coding oral cavity and oropharyngeal cancers have not changed significantly over time (Chaturvedi *et al*, 2008). We observed distinct birth cohort effects for both potentially HPV-associated oropharyngeal cancers and comparison site cancers, suggesting that the exposures within the underlying populations have changed over time. The decline in the incidence of comparison site cancer during the study period and within birth cohorts may reflect trends in smoking prevalence. In Australia, smoking prevalence was highest in males during the 1930s and 1940s at over 70% (Scollo and Winstanley, 2008) and has declined in subsequent years to a prevalence among adults of about 17% in 2007 (Australian Institute of Health and Welfare, 2008b). Although alcohol is also an established risk factor for oral cavity cancers (Blot *et al*, 1988; Franceschi *et al*, 1990; Hashibe *et al*, 2007), alcohol consumption in Australia last century more than doubled to peak in the 1970s and has since remained stable at a per capita consumption in those age 15 and over of between 9 and 10 l per year (Chikritzhs *et al*, 2003; Australian Bureau of Statistics, 2010). Although 83% of Australians are drinkers, almost 10% drink at levels posing a risk of long term harm (Australian Institute of Health and Welfare, 2008a), but its role in the incidence of comparison site cancer is unclear (McMichael, 1979).

We hypothesise that changes in sexual behaviour are contributing to the increasing incidence of potentially HPV-associated oropharyngeal cancers in recent birth cohorts. Previous studies have reported associations between sexual risk behaviour and cancers, which are associated with an increasing number of lifetime sexual partners (Heck *et al*, 2010), increasing numbers of oral sex partners (Gillison *et al*, 2008; Heck *et al*, 2010), early age at sexual debut (Heck *et al*, 2010) and among men with a history of same sex sexual contact (Heck *et al*, 2010). Australian sexual behaviour data show that the age of sexual debut has declined significantly from 19 and 18 among 50- to 59-year-old females and males, respectively, in 2001 to 16 among 16 to19 year old females and males (de Visser *et al*, 2003); the number of oral sex partners has been increasing (Rissel *et al*, 2003). Our observations of increased incidence of potentially HPV-associated oropharyngeal cancers among recent birth cohorts are consistent with this changing sexual behaviour. However, whether the predominance of these cancers in males is due to differences in sexual behaviour or biology is unknown (Marur *et al*, 2010).

This study has limitations that are relevant to its interpretation. First, our classification of oral cavity and oropharyngeal cancers as potentially HPV associated or comparison site was based on aetiological evidence from previous studies and not on an actual assessment of the tumour status for the presence of HPV DNA. Second, we were unable to collect data on tobacco and alcohol use among cancer cases and as a result, could not analyse the role of these risk factors. Third, we obtained data that had been collected and coded by eight different state and territory registries and collated by the NCSCH, and it is unclear whether each registry used the same method for handling sub-site cancers. However, given that they use the IARC and the International Association of Cancer Registries rules to code cancers, this is unlikely to be a significant problem (Fritz *et al*, 2000; IARC, 2004). Finally, some of our sub-group analyses by specific cancer type and for females were limited by the small numbers of cancer cases. Nevertheless, the trends observed for potentially HPV-associated oropharyngeal cancers were similar between males and females. Further, given that the prevalence of tobacco smoking among females increased during the 1960s and 1970s (Scollo and Winstanley, 2008), it will take some time before any potential decline in comparison site cancers, similar to that which has been observed in males, is also observed in females in Australia.

Given the role HPV has in the aetiology of some oral cavity and oropharyngeal cancers, HPV vaccines may be able to reduce the incidence of these cancers (Grulich *et al*, 2010). However, the natural history of HPV infection in the oral cavity and oropharynx remains unclear, with varying estimates of prevalence and an absence of a standardised collection and testing methodology (D’Souza *et al*, 2009; Kreimer, 2009; Syrjanen, 2010). If, as epidemiological data strongly suggests, genital–oral contact is an important route of HPV16 transmission into the oropharynx, then a falling exposure to genital HPV infection due to widespread vaccination may reduce the likelihood of HPV16 transmission, the risk of subsequent persistence and eventually cancer of the oropharynx. In addition, there is evidence that IgG antibodies produced by HPV vaccination transude into the oral cavity, potentially providing direct protection to vaccines (Rowhani-Rahbar *et al*, 2009). Our ability to predict the possible affect of prophylactic HPV vaccines on oropharyngeal cancers with any certainty awaits a better understanding of the epidemiology of HPV infection and its natural history in the oropharynx.

We found that while the incidence and proportion of comparison site cancers decreased during the period 1982–2005, the incidence and the proportion of potentially HPV-associated oropharyngeal cancers increased, particularly among recent birth cohorts. This increase may be attributable in part to changing sexual behaviours. Given that, it is likely that the incidence of HPV-associated oral cavity and oropharyngeal cancers will continue to increase, based on observed trends, the current HPV vaccination programme in Australia for females aged 12 to 13 years introduced in 2007 (Shefer *et al*, 2008) may have an affect on the future incidence of these cancers. The extent of this needs to be carefully modeled so that we can estimate the potential benefit from any further expansion of the current HPV vaccination programmes, including extending the programme to males.

## Figures and Tables

**Table 1:** Age standardised incidence rates of oropharyngeal and oral cavity cancers in Australia^a^

	**Potentially HPV-associated cancers**											
	**Tonsil** b			**Base of tongue and lingual tonsil** c			**Other oropharynx** d			**Comparison cancers** e		
**Time**	**Count (%)**	**Rate** f	**95% CI**	**Count (%)**	**Rate**	**95% CI**	**Count (%)**	**Rate**	**95% CI**	**Count (%)**	**Rate**	**95% CI**
*1982–2005*												
Total	3898 (100)	0.98	0.95–1.01	2610 (100)	0.66	0.63–0.68	2336 (100)	0.58	0.56–0.61	28 379 (100)	7.12	7.04–7.21
Females	925 (23.7)	0.45	0.42–0.48	623 (23.9)	0.30	0.27–0.32	493 (21.1)	0.23	0.21–0.25	6591 (23.2)	3.11	3.03–3.18
Males	2973 (76.3)	1.54	1.49–1.60	1987 (76.1)	1.04	0.99–1.08	1843 (78.9)	0.97	0.92–1.01	21 788 (76.8)	11.58	11.42–11.73
												
*2000–2005*												
Total	1315 (100)	1.09	1.03–1.15	954 (100)	0.79	0.74–0.84	661 (100)	0.55	0.50–0.59	7453 (100)	6.17	6.03–6.31
Females	282 (21.4)	0.46	0.40–0.51	227 (23.8)	0.36	0.31–0.41	128 (19.3)	0.20	0.16–0.23	1866 (25.0)	2.89	2.76–3.03
Males	1033 (78.6)	1.74	1.64–1.85	727 (76.2)	1.23	1.14–1.32	533 (80.6)	0.91	0.84–0.99	5587 (75.0)	9.75	9.5–10.01
												
*1994–1999*												
Total	993 (100)	0.95	0.89–1.01	786 (100)	0.75	0.70–0.80	650 (100)	0.62	0.57–0.67	7296 (100)	6.99	6.83–7.15
Females	236 (23.8)	0.44	0.38–0.49	190 (24.2)	0.35	0.30–0.39	149 (22.9)	0.27	0.22–0.31	1803 (24.7)	3.23	3.08–3.38
Males	757 (76.2)	1.50	1.39–1.61	596 (75.8)	1.19	1.09–1.28	501 (77.1)	1.02	0.93–1.11	5493 (75.3)	11.16	10.86–11.45
												
*1988–1993*												
Total	833 (100)	0.90	0.84–0.97	518 (100)	0.57	0.52–0.62	558 (100)	0.60	0.55–0.65	7114 (100)	7.70	7.52–7.88
Females	202 (24.3)	0.42	0.36–0.48	128 (24.7)	0.26	0.22–0.31	122 (21.9)	0.24	0.20–0.29	1559 (21.9)	3.18	3.02–3.33
Males	631 (75.7)	1.42	1.31–1.54	390 (75.3)	0.90	0.80–0.99	436 (78.1)	0.98	0.88–1.07	5555 (78.1)	12.72	12.37–13.06
												
*1982–1987*												
Total	757 (100)	0.92	0.85–0.98	352 (100)	0.43	0.39–0.48	467 (100)	0.57	0.52–0.62	6516 (100)	8.02	7.82–8.22
Females	205 (27.1)	0.48	0.41–0.55	78 (22.2)	0.18	0.14–0.22	94 (20.1)	0.22	0.17–0.26	1363 (20.9)	3.17	3.00–3.34
Males	552 (72.9)	1.41	1.29–1.53	274 (77.8)	0.71	0.62–0.79	373 (79.9)	0.97	0.86–1.07	5153 (79.1)	13.50	13.11–13.86

Abbreviations: CI=confidence interval; HPV=human papillomavirus.

a Squamous cell carcinomas.

b Tonsil (including Waldeyer's ring) – C09.0, C09.1, C09.8, C09.9 and C14.2.

c Base of tongue and lingual tonsil – C01.9 and C02.4.

d Other oropharynx – C02.8, C10.2, C10.8, C10.9, C14.0 and C14.8.

e Comparison cancers (Ryerson *et al*, 2008) –

(a) Oral tongue – C02.0, C02.1, C02.2, C02.3 and C02.9.

(b) Other oral cavity sites – C03.0, C03.1, C03.9, C04.0, C04.1, C04.8, C04.9, C05.0, C06.0, C06.1, C06.2, C06.8 and C06.9.

(c) Larynx – C32.0, C32.1, C32.2, C32.3, C32.8 and C32.9.

(d) Other oropharynx sites – C05.1, C05.2, C05.8, C05.9, C10.0, C10.1 and C10.3.

f Rates are per 100 000 population and are age standardised to the 2001 Australian population (5 year age groups). (Australian Bureau of Statistics, 2008.)

**Table 2:** Age at diagnosis of oral cavity and oropharyngeal cancers (2005), trends in mean age over time (1982–2005) and changes in age standardised incidence over time (1982–2005)^a^

		**Cancers diagnosed in 2005**		**Trends in age at diagnosis from 1982 to 2005**		**Changes in incidence from 1982 to 2005**	
**Sex**	**Cancer site**	**Count**	**Mean age at diagnosis (95% CI)**	**Percentage change per calendar year (95% CI)** b	***P*-value**	**Percentage change per calendar year (95% CI)** c	***P*-value**
Males	Potentially HPV associated	413	59.8 (58.8–60.9)	−0.06 (−0.11, −0.01)	0.02	1.42 (1.08 to 1.76)	<0.01
	Tonsil	193	57.4 (56.0–58.9)	−0.18 (−0.24, −0.11)	<0.01	1.39 (0.88 to 1.92)	<0.01
	Base of tongue	125	60.3 (58.5–62.2)	0.02 (−0.05, 0.09	0.54	3.02 (2.27 to 3.78)	<0.01
	Other oropharynx	95	64.1 (61.8–66.4)	0.09 (−0.01, 0.18)	0.06	−0.19 (−0.95 to 0.59)	0.64
	Comparison	994	64.4 (63.7–65.2)	0.12 (0.10, 0.15)	<0.01	−1.69 (−1.96 to −1.42)	<0.01
Females	Potentially HPV associated	118	63.6 (61.6–65.6)	0.04 (−0.07, 0.14)	0.47	1.04 (0.40 to 1.68)	<0.01
	Tonsil	52	63.1 (60.2–66.1)	−0.03 (−0.17, 0.10)	0.61	0.12 (−0.95 to 1.19)	0.83
	Base of tongue	43	62.9 (59.5–66.3)	−0.01 (−0.15, 0.14)	0.93	3.45 (2.21 to 4.70)	<0.01
	Other oropharynx	23	65.8 (60.8–70.7)	0.20 (0.02, 0.39)	0.03	−0.24 (−1.57 to 1.10)	0.72
	Comparison	324	66.9 (65.4–68.5)	0.07 (0.03, 0.12)	<0.01	−0.39 (−0.81 to 0.03)	0.07

Abbreviations: CI=confidence interval; HPV=human papillomavirus.

a Squamous cell carcinomas.

b Estimated by linear regression analysis.

c Estimated using generalised linear models with a logarithmic link function.
